# Across the spectrum: integrating multidimensional metal analytics for *in situ* metallomic imaging

**DOI:** 10.1039/c8mt00235e

**Published:** 2018-11-30

**Authors:** Theodora J. Stewart

**Affiliations:** a King's College London , Mass Spectrometry , London Metallomics Facility , 4th Floor Franklin-Wilkins Building , 150 Stamford St. , London SE1 9NH , UK . Email: theodora.stewart@kcl.ac.uk; b King's College London , School of Biomedical Engineering and Imaging Sciences , 4th Floor Lambeth Wing, St. Thomas’ Hospital , London SE1 7EH , UK

## Abstract

To know how much of a metal species is in a particular location within a biological context at any given time is essential for understanding the intricate roles of metals in biology and is the fundamental question upon which the field of metallomics was born. Simply put, seeing is powerful. With the combination of spectroscopy and microscopy, we can now see metals within complex biological matrices complemented by information about associated molecules and their structures. With the addition of mass spectrometry and particle beam based techniques, the field of view grows to cover greater sensitivities and spatial resolutions, addressing structural, functional and quantitative metallomic questions from the atomic level to whole body processes. In this perspective, I present a paradigm shift in the way we relate to and integrate current and developing metallomic analytics, highlighting both familiar and perhaps less well-known state of the art techniques for *in situ* metallomic imaging, specific biological applications, and their use in correlative studies. There is a genuine need to abandon scientific silos and, through the establishment of a metallomic scientific platform for further development of multidimensional analytics for *in situ* metallomic imaging, we have an incredible opportunity to enhance the field of metallomics and demonstrate how discovery research can be done more effectively.

## Abstract 2

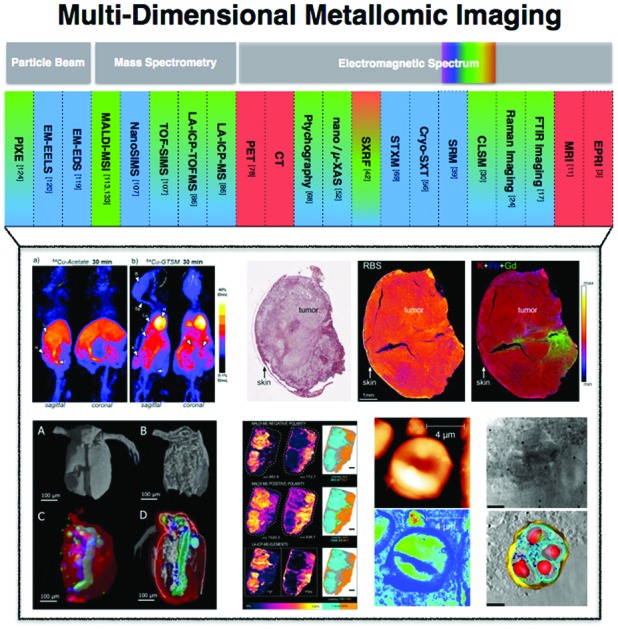
Taking a systems analytical approach to systems biology questions requires a network of multidimensional analytical tools to illuminate the many different functional and structural aspects of metals in biology.

## Introduction

I often hear the phrase “there is no silver bullet” when it comes to metal analytics. Have a conversation with any analytical chemist and the multitude of limitations each technique inherently possesses shall be quickly revealed. As disheartening as this may be, I cannot argue with the obvious analytical challenges and fully realize the necessity of utilizing a portfolio of complementary techniques. Yet, from my own personal experience and speaking to researchers coming from a wide range of scientific disciplines, it is also clear that we are not even fully aware of how many “bullets” exist.

We all have, at the very least, an intuitive sense that the functions of essential metals in biology are complex, dynamic and critical for life. Nature possesses a whole arsenal of diverse approaches to support such processes. Therefore, to reveal Nature's secrets we must approach metallomic studies with an equally rich toolkit of integrated analytics across multiple dimensions of space and time, which are specific, sensitive and flexible enough to accommodate the wide range of concentrations, complex chemistry, and dynamics of metals in biology. This article is not merely another review of analytical techniques, of which there are plenty of informative works. In this perspective piece, I present a different way to relate to and integrate the analytics we use, placing a specific focus on current and emerging techniques for *in situ* metallomic imaging. Seeing is believing, and a picture is worth a thousand words. In the following pages, I take us on a journey through a network of multidimensional analytical tools, using the electromagnetic spectrum as a platform, moving from microwaves to gamma rays, then into the worlds of mass spectrometry and particle based imaging techniques to understand how the interaction between energy and living matter can illuminate so many different functional and structural aspects, which we require as scientists to comprehend the rich world of metals in biology.

**Fig. 1:**
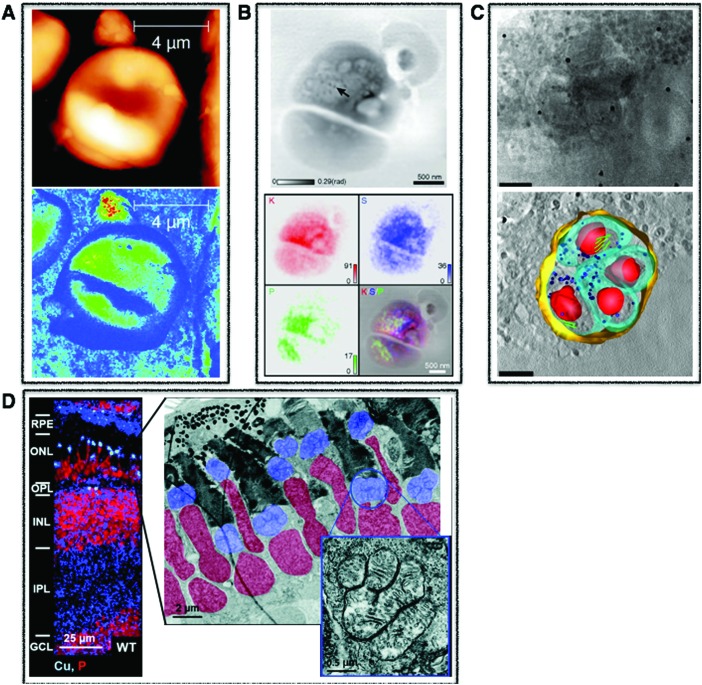
Cellular multimodal imaging. (A) AFM scan of a single red blood cell (upper) with parallel s-SNOM subcellular chemical contrast image (67 nm resolution) (lower). Adapted from Amrania *et al.*^20^ (B) Ptychographic image of frozen-hydrated *Ostreococcus* alga with arrow poiting to ribosome-like complexes (upper) and corresponding XRF maps of potassium (red), sulphur (blue) and phosphorus (green) with subsequent overlay (lower). Scale bars are 500 nm. Adapted from Deng *et al.*^72^ (C) Cryo-SXT image of a human fibroblast cell vacuole containing *Toxoplasma gondii* (upper) and the reconstructed volume showing the parasitophorous vacuole (yellow) with four parasites and their respective plasma membranes (cyan) rhoptries (green) and nuclei (red). Scale bars are 2.5 mm. Adapted from Harkiolaki *et al.*^57^ (D) NanoSIMS copper (blue) and phosphorous (red) overlay of wt zebrafish retina (left) and electron micrograph of similar region (right) with highlighted nuclei (red) and megamitochondria (blue) with inset corresponds to one megamitochondrion, indicating co-localisation of copper puncta and megamitochondria. Scale bars are 25 μm, 2 μm, and 0.5 μm, respectively. Adapted from Ackerman *et al.*^135^

**Fig. 2:**
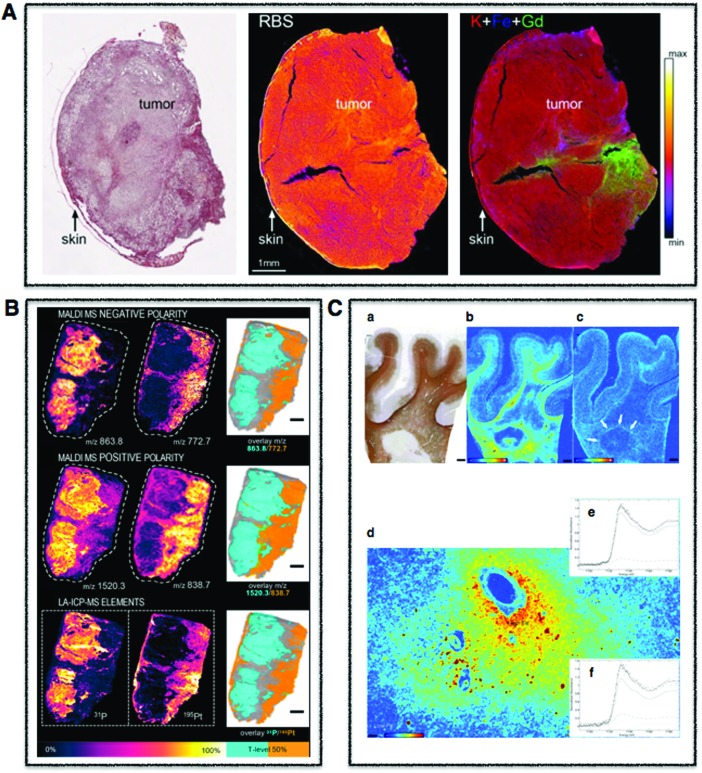
Tissue multimodal imaging. (A) Histological section of U87 human glioblastoma xenograft (left), RBS image of tissue section (middle), PIXE image of tissue section highlighting potassium (red), iron (blue), and gadolinium (green) corresponding to injected gadolinium NPs (right). Scale bar 1 mm. Adapted from Carmona *et al.*^129^ (B) Sections of human malignant pleural mesothelioma treated with cisplatin highlighting distribution of specifc phospholipids using MALDI-MSI in negative (*m*/*z* 772.7 and 863.8) (upper) and positive (838.7 and 1520.3) (middle) modes. LA-ICP-MS was used to map distribution of phosphorus and platinum. Scale bar 1 mm. Adapted from Holzlechner *et al.*^136^ (C) Histological section of apparently normal and periplaque white matter of multiple sclerosis lesion in human brain (a) and corresponding iron (b) and zinc (c) XRF maps. Scale bars 3 mm. Iron shown to accumulates perivascularly in astrocytes (d) and quantitative speciation of iron in concentrated regions (e) and in iron-poor regions (f). Scale bar 90 μm. Adapted from Popescu *et al.* under the Creative Commons Attribution 4.0 International License (; http://creativecommons.org/licenses/by/4.0/).^53^

**Fig. 3:**
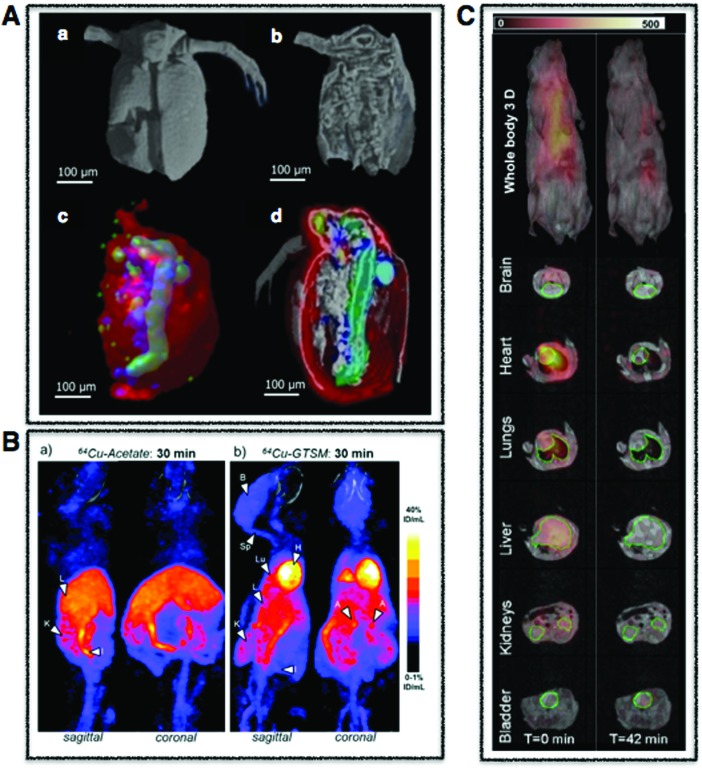
Whole body multimodal imaging. (A) μ-CT 3D renderings of *C. dubia* (a and b) and calcium (red), manganese (green), zinc (blue) isosurfaces using confocal μ-XRF (c and d). Scale bar is 100 μm. Adapted from Van Malderen *et al.*^102^ (B) PET/CT maximum intensity projections (MIPs) of 6–8-month-old wild-type mice at 30 min post-injection of Cu-acetate (a) and Cu-GTSM illustrating different uptake patterns. Adapted from Andreozzi *et al.*^84^ (C) Superimposed 3D EPRI and 3D proton MRI images in whole body and specific organs in cigarette exposed mice, where intensity distribution corresponds to EPR intensity of 3-CP nitroxide probe distribution at time 0 and 42 min. Adapted from Caia *et al.*^137^

Metallomics is powerful in that it presents an opportunity to shift our scientific thinking, approach and methodologies to more comprehensively understand our system of study, offering integration of existing research disciplines at a higher level. This same approach can be taken when relating to metallomic analytics. Ironically, the phase “metallomic analytics” is, in fact, an oxymoron. The word analysis originates from the ancient Greek word 
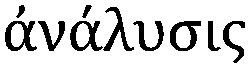
 from *ana*- “up, throughout” and *lysis* “a loosening,” literally implying a breaking up, in seeming contradiction with metallomics, a bottom up synthesis approach to understanding metals in biology. How then can we use an approach that is inherently disruptive within the context of a field, which is inherently integrative? Rather than picking a few familiar techniques and operating within their analytical confines, what if we viewed our analytical possibilities in an integrated and comprehensive context? *A systems analytical approach to systems biology questions*, where just as in Nature, the whole is much more than the sum of its individual parts, demarcating a shift in the way we utilise analytical chemistry and physics from a series of separated individual tools to probe a living system towards an integrated network of analytics as powerful as the living biology it is used to study. The goal in integrating current techniques, whilst continuing further analytical developments directly informed by biological scientific needs, is to ultimately understand the role of a specific metal, at a specific location in time within the context of a cellular process and how those dynamic functions cascade to higher levels of function at the tissue, organ and organism levels. In the following sections, I discuss analytical techniques, which allow us to identify, quantify and directly probe the immediate surrounding chemical environment (oxidation state, local coordination geometry) of a metal, its localisation in both two and three dimensions at the cellular (Fig. 1), tissue (Fig. 2) and organ (Fig. 3) levels, and even assess temporal dynamics under certain conditions.

## Spectroscopy imaging

**Fig. 4:**
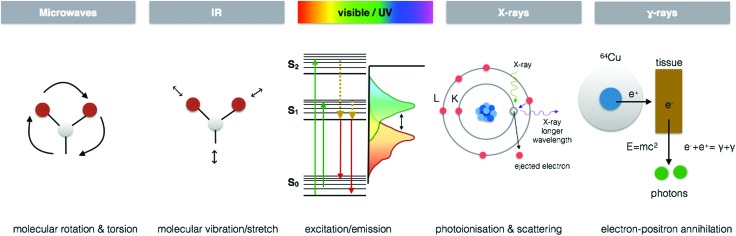
Energy–matter interactions of the electromagnetic spectrum. Spectroscopic imaging techniques arise from fundamental energy–matter interactions. EPRI exploits the molecular rotation and torsion caused by microwaves to detect paramagnetic species by measuring the magnetism of electrons and their change when bound to molecular structures. FTIR and Raman imaging take advantage of characteristic vibrations/stretching and even rotation associated with specific molecular species when interacting with infra-red for FTIR and typically extending to near infra-red and the visible region for Raman. CLSM and SRM utilise characteristic fluorophore excitation/emission profiles in the UV/vis region, exploiting the Stokes shift that occurs through loss of vibrational energy between absorption and emission to localise multiple fluorescent signals at the subcellular level. X-ray spectroscopic techniques benefit from element specific photoionisation energies and subsequent scattering of electrons to reveal detailed electronic structural information at the atomic scale. PET utilises gamma-ray emission that results from the annihilation of electrons in tissue from positron emitting isotopes.

Each region of the electromagnetic spectrum possesses unique physical properties, which can be harnessed and utilised in analytical tools to gain information from the interaction of electromagnetic radiation and matter on questions pertaining to metal functions in biological processes (Fig. 4). When combined with optical, electron, or scanning probe microscopes, these spectroscopic signals are amplified to visualize structural and chemical information invisible to the naked eye. In the following section, I present several key spectroscopy imaging techniques for *in situ* metallomic studies ranging from whole body to subcellular imaging and highlight particular advances in both optical and X-ray microscopies that have been key in going beyond diffraction limitations to image at the nanoscale.

### Electron paramagnetic resonance imaging (EPRI)

Electron paramagnetic resonance (EPR), also referred to as electron spin resonance (ESR), assesses the electronic structure of molecules through measuring the magnetism of unpaired electrons in an external magnetic field. Within the context of metallomics, EPR can be used to obtain detailed information on the coordination chemistry specifically of paramagnetic species (species with unpaired electrons), which include free radicals and some transition metal complexes in biological samples. For example, Mn^2+^ complexed by specific ligands at low temperature shows a significantly different spectrum relative to the free ion Mn^2+^.^1^ Although limited to paramagnetic and ferromagnetic species, conveniently these species are common intermediates in redox reactions found in both normal and aberrant metabolism, and transition metal complexes constitute the active centres of bio-macromolecules. EPR plays a particularly important role in elucidating mechanisms of metal redox chemistry.

Traditionally, EPR has been used to characterize metal centres in both isolated and purified proteins and model compounds.^2^ However, EPR has probably gained its widest biological potential yet as EPR Imaging (EPRI) within the context of biomedical research.^3^ With EPRI, imaging of tissue O_2_ partial pressure (*p*O_2_)^4^ and reactive oxygen species (ROS) microenvironments is possible and ideal in the sense that it is both non-invasive and quantitative so that it can be carried out as an *in vivo* technique (Fig. 3C).^5^ By using a large range of commercially available paramagnetic probes, *p*O_2_ can be imaged and quantified, and by using a technique called spin-trapping, ROS can be imaged and followed over time.^6^ Unfortunately, EPRI does not provide anatomical information and suffers from poor spatial resolution (1 mm), relative to other imaging techniques. Even with this limited spatial resolution, EPRI has been used to map redox status in tumours, which can been used to predict resistance and reoccurrence to radiation therapies.^4^,^7^ When combined with other metal imaging modalities, EPRI can provide an important chemical context for tissue level metal redox processes involving unpaired electrons.

### Magnetic resonance imaging (MRI)

The physical context is often critical for metallomic questions: which subcellular compartment or where in a tissue is the metal of interest? Developed as a tool to provide anatomic images, MRI has provided that contextual foundation during the course of its evolution over the past four decades. Paramagnetic metal ions and superparamagnetic iron oxide nanoparticles are often used as contrast agents, and greater refined metal-based complexes (*i.e.* Gd(iii), Lu(iii), Eu(iii)) are now being used as reporter molecules for multimodal imaging approaches, combing the properties of MR contrast with fluorescence and possessing even targeting capabilities.^8^

Typical resolutions for *in vivo* MRI fall around 1 mm^3^, however advances in both molecular and cellular MR imaging (MR-microscopy (MRM)) now allow for longitudinal monitoring of subcellular events whilst providing highly resolved anatomical contexts with spatial resolutions in the sub 100 μm range. MRM has been used to reveal characteristic pathologic features of both benign and malignant breast and lymph tissue,^9^ and as an *in vitro* tool to study myonuclei of individual muscle fibres down to 6 μm planar spatial resolution.^10^ Particularly relevant for metallomics, MRI chemical probes have also been synthesised for specific investigations of biologically important metal ions, such as potassium, magnesium, copper(i), copper(ii), zinc, calcium and even toxic metals such as lead and cadmium.^11^ In the case of zinc, the Zn^2+^ sensor GdDOTA-diBPEN has been successfully used to image and quantify labile (non-protein bound) Zn^2+^ in the μM concentration range in mice within the context of zinc release in functional β-cells in the pancreas *in vivo* and, when administrated to type-1 diabetes mouse model, was sensitive to β-cell loss of function, detecting decreased release of Zn^2+^.^12^ The same probe has also been used to detect differences in healthy and tumorigenic prostate in mice, showing reduced concentrations in cancerous prostates, suggesting a possible diagnostic tool.^13^ With the help of metal specific MRI probes, an exciting era of research is emerging for *in vivo* deep tissue monitoring of metals.

### Fourier Transform infrared (FTIR) imaging

Moving across the electromagnetic spectrum to the infrared region we come to Fourier Transform infrared spectroscopy (FTIR), one of several types of vibrational spectroscopies that uses differential absorbance, transmittance or reflectance of polychromatic infrared radiation to provide information on characteristic vibrational frequencies of chemical bonds, providing a chemical fingerprint sensitive to hetero-nuclear functional group vibrations as well as polar bonds. In fact, although not explicitly integrated into the names of other imaging techniques, FT has become an integral signal-processing tool in many of the techniques discussed below. With its ability to break down complex wave-like signals to extract meaningful spectra, like taking a cake (waveform) and extracting the recipe (the FT), no wonder physicist Lord Kelvin described Fourier's theorem as “an indispensible instrument in the treatment of nearly every recondite question in modern physics.”^14^ FTIR is particularly sensitive to changes in secondary structure of proteins, making it an important tool in the study of protein aggregation and misfolding, which is often observed in combination with altered distribution of metals. When used as a micro-spectroscopic technique, FTIR imaging is able to generate label-free, molecular-specific images to visualize the distribution of different components (*i.e.* protein, lipid, carbohydrate, nucleic acids) within tissue sections.^15^ In this sense, FTIR imaging can be considered as a complementary metabolomic tool to metallomic inquiries that allows for the *in situ*, non-destructive analysis of biological specimens.^16^,^17^

To obtain biologically meaningful spatially resolved spectra in cells and tissues, the brilliance of a synchrotron source is often utilized to overcome challenges associated with small aggregates at the nanoscale level as well as to detect the often subtle changes in the FTIR spectra observed *in vivo*. By circularly accelerating electrons close to the speed of light to produce intense synchrotron light 10 billion times brighter than the sun, interactions at the finest atomic and molecular scales can be studied. Access to synchrotron facilities is free, but requires a peer-reviewed application process with calls typically every six months. Although access is limited, synchrotron radiation (SR) has proved to be an invaluable tool, measuring protein misfolding and aggregation in tissue in Alzheimer's, Parkinson's and Huntington's disease as well as amyotrophic lateral sclerosis and even scrapie.^18^ However, recent advances in scattering-type scanning near-field optical microscopy (s-SNOM), where near IR light is focused on a sample through the probe of an atomic force microscope (AFM), routinely allows for measurements at 10 nm and down to 1 nm.^19^ This principle has just been extended to cover the mid IR region, or the important “fingerprint” region, where a single red blood cell at 67 nm spatial resolution was recently imaged (Fig. 1A).^20^ When combined with techniques directly mapping metal distributions, FTIR can provide important information about the compositional environment of a metal at both the tissue^21^ and now cellular levels in two and three dimensions.^22^

### Raman imaging

Raman spectroscopy also probes vibrational modes of molecules and is based on the similar principles underpinning FTIR vibrational spectroscopy, but differs in several fundamental ways by utilising monochromatic light (rather than polychromatic) to measure change in the polarizability (rather than dipole moment) of a molecule. Raman is sensitive to homo-nuclear molecular bonds and can distinguish between C–C, C=C and C≡C bonds. Therefore, what is often “invisible” to FTIR is measured in Raman and *vice versa*. Raman has become a valuable tool for filling a gap through its ability to detect small structural changes and complementing other techniques like X-ray crystallography, which provide a larger structural picture.^23^ Although Raman is highly molecularly selective, it suffers from low sensitivity, often competing with the strong auto fluorescence generated from biological samples. However, specific types of Raman, primarily resonance Raman spectroscopy (RRS) and surface enhanced Raman spectroscopy (SERS), can be used, as signals are inherently enhanced.

Just as in FTIR, Raman can be used as an imaging tool, providing morphological as well as both qualitative and quantitative biochemical information at subcellular resolution in living cells.^24^ In resonance raman spectroscopy (RRS), enhancement of specific chromophores are used, the most popular being π → π* transitions from conjugated π-bonds. Fortunately within the context of metallomics questions, these conjugated π systems are associated with critical cellular processes involving metals, as in the case of porphyrin derivatives, which constitute a main part of enzymatic cofactors. One such example is the enzyme cytochrome *c*, an iron containing heme protein essential in the respiratory system of eukaryotic organisms. Using RRS, the localisation of both the reduced (Fe^2+^) and oxidized (Fe^3+^) forms of cytochrome *c* and *b* in living murine cells were quantitatively determined, revealing that both cytochromes are co-distributed, but that cytochrome *c* is predominantly reduced whereas cytochrome *b* is predominantly oxidized.^25^ Obtaining this degree of chemical information in living cells at subcellular resolution presents exciting opportunities and a powerful context for specifically studying mitochondrial metal metabolism, which when disrupted, has been implied in a wide range of human diseases including red blood cell disorders, cardiomyopathy, skeletal myopathy, and neurodegeneration.^26^ Raman studies have also been used to characterize cancer tissues,^27^ which when combined with spatially resolved metal analyses have the potential to yield an even stronger identifying “fingerprint”. The reader is directed further to a recent detailed review of the different types of Raman spectroscopy and their state of the art applications in biology.^28^ Although advancements towards high-resolution spectroscopy for Raman have been made as illustrated with FTIR, the low sensitivity of Raman still requires larger laser power for tip-enhanced Raman, and therefore problematic for delicate biological samples. However, recent advances in spectroscopic stimulated Raman scattering (SRS) now allow for real-time metabolic imaging at the subcellular scale.^29^ Like in FTIR, Raman imaging provides a “molecular fingerprint” from which detailed information can be obtained on the local sites within larger macromolecules for metallomic studies.

### Light microscopy

We often say that seeing is believing, and no other scientific technique underpins this statement more than light microscopy. There is an excitement and intuitive pull in moving beyond the inherent limitations of the human eye to see life at more subtle and powerful scales. Starting with the humble beginnings of light microscopy in 1665, marked by Hooke's manuscript “Micrographia” and pioneering work by van Leeuwenhoek, the inconvenient laws of physics have seemed to always present a barrier to witnessing the most fundamental processes of life at the subcellular scale. Even within the confines of that pesky 250 nm diffraction limit, significant advances in light microscopy have led from the evolution of transmission bright field to confocal laser scanning microscopy, which has revolutionised the field of biology in the past 20 years. With recent advancements in optics, electronics and mathematics we are finally able to overcome the diffraction limit, giving rise to super resolution microscopy, again shedding new light on cellular processes now at the molecular level. What sets light microscopy apart from techniques such as X-ray microscopy and mass spectrometry discussed later is the ability to non-destructively work with living samples, opening investigations into an additional dimension (4D) in time by capturing live cell dynamics.

#### Confocal laser scanning microscopy (CLSM)

Within the field of metallomics, visualising metals using CLSM with metal specific probes and sensors has dramatically shifted and elevated our understanding of the significance of essential metal homeostasis in cellular functions. Metal-sensitive fluorescent sensors, one of the most powerful and widely used tools in cellular metallobiology,^1^ are molecules that recognise and specifically, and often reversibly, bind metal ions.^30^ Upon such binding, a change in fluorescence intensity and/or emission wavelength occurs in either a switch on (stimulation) or a switch off (quenching) way that is correlated to the presence or concentration of the metal ion. Sensors are ideally both specific and selective, particularly when quantification is desired beyond imaging spatial distribution and cellular fluxes. Importantly, these sensors allow spectroscopically “invisible” ions such as Zn^2+^ and Ca^2+^ to be “seen”, revealing new functions of non-protein bound metals.^31^,^32^

The use of fluorescent sensors to measure intracellular metal ions has been extensively reviewed.^30^ In brief, sensors can be broadly classified into three main types: molecular, genetically encoded, and hybrid. Some of the more commonly used sensors are molecular probes, which involve small-molecule fluorophores coupled to a metal chelator and must be externally delivered into the cell. However, these sensors often lack control over subcellular localisation and are unable to detect very low concentrations of free ions, which can be biologically relevant for certain metal ions like Zn^2+^ and Cu^+^. Genetically encoded probes are synthesised within the cell and can be used to overcome these limitations through the use of fluorescent proteins and a peptide or protein moiety for metal binding. When two fluorescent proteins are used, Förster resonance energy transfer (FRET) can be exploited creating a powerful ratiometric sensor where metal binding can either promote or disrupt FRET. Hybrid probes are simply a combination of small-molecule and genetically encoded probes, enabling protein/peptide tags to be used for localising small molecule sensors to specific subcellular compartments. It is important to note that “not all metals are created equal” and their wide range of concentrations and chemical properties present specific challenges for fluorescence based detection of certain intracellular metals like copper. Unlike Ca^2+^ found in relatively high concentrations within the cell, low intracellular Cu^+^ and Cu^2+^ concentrations are exquisitely maintained, balancing essential yet potent redox activity with a propensity to exacerbate oxidative stress. Although a handful copper sensors have been developed, far fewer are available relative to Ca^2+^ or Zn^2+^, as Cu^+^ can easily oxidise to Cu^2+^ and Cu^0^ in water and quench fluorescence due to its redox properties.

The case of zinc is exemplary in how visualisation and quantification of a metal in space and time can alter our understanding of the role of essential metals in biological processes. Zinc is recognised as an essential micronutrient required for cellular homeostasis. However, until recently it was thought to only serve structural and catalytic functions in proteins and enzymes. With the ability to quantify intracellular changes down to pM of free Zn^2+^ thanks to advances in zinc specific probes and sensors,^33^–^35^ along with their use to highlight the roles of zinc in processes spanning from modulating tyrosine phosphatase 1B activity^36^ to the quantitative mapping of zinc sparks released during fertilisation required for egg to embryo transition,^37^ its critical role in cell signalling has finally been acknowledged, opening the field of zinc biology to new exciting research questions.

#### Super resolution microscopy (SRM)

The resolution achieved by CLSM has been sufficient to interrogate tissue morphology and even whole cell dynamics. However, the development of more sophisticated experimental techniques that allow us to gather information about complex and dynamic subcellular processes with a need to visualise such processes with greater resolution has propelled the microscopy field forward utilising new approaches referred to as super resolution microscopy (SRM). The most widely used SRM approaches include structured illumination microscopy (SIM), which when combined with selective plane illumination microscopy (SPIM) results in what is commonly referred to as light sheet microscopy, STimulated emission depletion microscopy (STED), and single molecule localisation microscopy (SMLM), which has two commonly used modalities: photo activated localisation microscopy (PALM) and stochastic optical reconstruction microscopy (STORM). Confusing acronyms aside, these techniques allow for imaging at the sub 100 nm scale in both two and three dimensions, and with lattice light sheet high imaging speeds of larger areas is now possible up to a few hundred frames per minute to acquire volumetric data (*i.e.* of entire HeLa cells every 4 s).^38^ As there is no silver bullet in science, each approach has its respective advantages and disadvantages. SMLM with the highest possible resolution is suited for co-localisation questions, whereas STED better suits dynamic processes. When simple sample preparation is required SIM presents an acceptable compromise between speed and resolution. The reader is directed to a recent excellent review for more detailed information on principles and applications of SRM.^39^ The application of metal sensors and probes using SRM techniques seems a logical step for application in metallomic studies. However, the specific fluorophore requirements for such techniques preclude many commonly used CLSM fluorophores from being directly translated to SRM, and is a likely explanation for the absence of metal specific SRM work presented in the literature. However, this also presents a unique opportunity to develop such metal specific probes, which may allow for the high-resolution study of dynamic metal trafficking at the subcellular scale. With SRM, we have entered an era where we can now study molecules with a microscope, capturing dynamic processes in 3D, leading to valuable contextual information within metallomic studies. However, to reach the finest structural resolutions we must venture into the realm of X-rays.

### X-Ray microscopy

Cellular processes involving metals are dictated by complex metal chemistry played out at the atomic and molecular scales. To glimpse into this nano world requires spectroscopic techniques utilising wavelengths powerful enough to excite and even ionise core electrons and at spatial scales corresponding to the Ångstrom distances found between interacting atoms. This level of detailed study requires X-rays, both scattering and refracting in their interaction with matter. By harnessing and taking advantage of these energy–matter interactions, X-ray spectroscopic techniques provide tools to probe local coordination geometry and chemical states of species as well as provide structural information at the subcellular level, thereby providing the unique advantage of determining metal speciation, a critical component in understanding the chemistry of metals in cellular processes. X-ray microscopy provides spatial resolutions down to tens of nm without the need for invasive approaches like labelling, dehydration, or chemical fixation, which can alter endogenous metal chemistry. The ability to obtain subcellular resolution is particularly important within the context of cellular metal chemistry, as metal homeostasis relies on tight regulation of metal species often through subcellular compartmentalisation. A few key examples include Fe–S clusters and heme synthesis products in the mitchochondria, zinc finger proteins required for gene transcription in the nucleus, and regulation of copper in the Golgi.^40^,^41^ Although the types of X-ray spectroscopies discussed are not limited to synchrotron radiation sources, in combination with optics allowing for sub-micron beam sizes, synchrotron radiation provides the high flux and brightness required for enhanced resolution and sensitivity required when imaging metals in biological samples at the subcellular level.

X-ray microscopy can be broadly grouped into techniques providing elemental identification and speciation and those providing structural and morphological information. Localisation and quantification of elements in cells and tissues can be achieved using X-ray fluorescence (XRF), and when combined with X-ray absorption spectroscopy (XAS), additional speciation information is obtained on the chemical state and local coordination environment. To place such compositional information into a structural context, absorption or phase contrast tomography can be utilised to obtain information on cellular structure in both two and three dimensions, while X-ray diffraction techniques provide more detailed structural information and probe the local nanostructure and chemical composition of a sample. The most relevant X-ray microscopy methods for *in situ* metallomics along with examples are described below.

#### Synchrotron X-ray fluorescence (SXRF) microscopy

Each element possesses electronic orbitals with associated characteristic energies, and XRF microscopy takes advantage of these element-specific electronic structures. Incident photons with enough energy above the ionisation threshold eject a core electron, which is subsequently filled by an outer shell electron resulting in the emission of a lower energy fluorescent photon. When these measured element specific transitions are combined with X-ray beams rastered across the surface of a sample, quantitative elemental distribution maps with corresponding spatial resolutions in the μm to nm range are produced using μ-XRF and nano-XRF, respectively (Fig. 1B and 2C). When combined with tomography, 3D elemental distribution maps can be generated^42^ (Fig. 3A), and for important transition metals such as iron, nano-XRF has shown absolute detection limits on the order of 10^–18^ g at the subcellular level (90 nm).^43^

A wide range of metallomic questions have utilised both μ-XRF and nano-XRF, including questions regarding non-essential metal detoxification and effects to endogenous metals at the subcellular level, metal profiles in cellular processes and human disease, and subcellular localisation of metallo-based drugs, to highlight just a few examples. It was shown that silver and cobalt taken up in the unicellular algae *Coccomyxa actinabiotis* does not impact endogenous metal distributions, and that silver is taken up and seemingly sequestered in vacuoles, whereas cobalt is homogenously found directly outside the chloroplast.^44^ Intriguing work on the inheritance of trace metals during mitosis in NIH 3T3 mouse fibroblast cells showed the spatial dynamics of copper and zinc during metaphase, a 3-fold increase of zinc during interphase, and then resulting pairs of twin metal pools during cytokinesis, all indicating a more important physiological role of zinc than previously thought.^45^ Work regarding selenium-mediated arsenic hepatobiliary excretion in mammals used μ-XRF to co-localise arsenic and selenium in the liver, gall bladder and small intestine of hamsters.^46^ Metal dyshomeostasis is thought to be involved in many human diseases including neurodegenerative disease.^47^ Using μ-XRF it was shown that both iron and calcium increase in the brain of both control and Alzheimer's disease (AD) mice, and that copper, iron, zinc and calcium are all significantly higher in AD mice relative to controls indicating a possible role of metal dyshomeostasis in AD.^48^ Primary cultured neurons, derived from rat embryonic cortex (CTX) and two regions of the hippocampus, displayed characteristic spatial metal signatures (calcium, iron, zinc, copper, manganese), concluding that metal distribution in the brain is likely a result of not only extrinsic factors related to synaptic function, but also intrinsic factors controlling cellular metal content and distribution.^49^ Moving to the nano scale, nM concentration of an osmium based anti cancer drug were mapped in whole ovarian cancer cells with 50 nm spatial resolution, finally being able to resolve subcellular organelles and showing that osmium localises to the mitochondria not the nucleus, along with the mobilisation of calcium from the endoplasmic reticulum, signalling cell death.^50^ SXRF is the most sensitive of the X-ray based techniques, on par with that of mass spectrometry imaging based techniques (discussed later) and shown to be even more sensitive at the highest spatial resolutions.^51^ It is not surprising that to date, SXRF is considered to be the most reliable approach for quantitative and non-destructive imaging of elemental distributions at the sub-micron scale.

#### X-Ray absorption spectroscopy (XAS)

For certain biological questions, not only are the total metal distributions obtained from SXRF important, but also the speciation of metals in specific subcellular regions, as chemical speciation influences function. Using XAS, information on elemental speciation can be obtained taking advantage of an element-specific feature called an absorption edge, which arises when the incident X-ray energy corresponds to the binding energy of a core electron of a specific element of interest. The region around the absorption edge is referred to as the X-ray absorption near edge structure (XANES) and can be thought of as a fingerprint region. For instance, it can be used to distinguish valence states, *e.g.* Cu^+^ and Cu^2+^ in biology. When the incident energy provides enough kinetic energy for a core electron to leave the atom, a core electron is ejected and scattered by surrounding atoms, where the outgoing and backscattered waves constructively or destructively interfere and result in what is referred to as the extended X-ray absorption fine structure (EXAFS). The XANES region, providing information on the oxidation state and local geometry, is typically used as a fingerprinting tool and most commonly used in XAS spatial mapping,^52^ while the EXAFS region provides additional information about the geometry of the complex and the type of ligands.

XAS mapping is used to determine speciation of metals in biological tissues and now possible at the cellular level with both scanning transmission X-ray microscopy (STXM) and the more recent introduction of hard X-ray nanoprobes. Determination of oxidation states and speciation “fingerprinting” through the spectral comparison with biologically relevant standards of metal complexes can be used to decipher the metal speciation in biological samples. Many of the SXRF examples previously highlighted were accompanied with XAS for metal speciation determination. For example, using XAS, the co-localisation of selenium and arsenic in liver was determined to be the seleno bis-(*S*-glutathionyl) arsinium ion, which was excreted *via* bile into the intestinal tract, thus supporting a previous hypothesis of hepatobiliar excretion.^46^ In the Alzheimer's Disease (AD) work, XANES showed evidence of copper redox properties in AD mouse brains,^48^ and accumulation and storage of ferritin in reactive astrocytes within smouldering and inactive plaques of Multiple Sclerosis (MS) patients were discovered using XANES (Fig. 2C).^53^ In work providing greater insight into the role of bromium in biological systems, it was shown that bromium is widely distributed across a range of mammalian tissues and fluids and that the major form is bromide, where it was seen in elevated concentrations in the sub-endothelial regions of arterioles in bovine ovaries.^54^ STXM is particularly unique in that it utilises X-rays extending into the soft-X-ray regime, allowing for speciation of elements such as carbon, nitrogen, and oxygen but also includes biologically important transition metals like iron and manganese. Because of this capability, certain transition metals can be speciated within the context of proteins, lipids, polysaccharides, and nucleic acids mapped down to 20 nm at certain synchrotron beam lines and can be done in 3D if proper optical configurations are present. The majority of work conducted has been within the context of biofilms and mineralizing bacteria.^55^ Utilising the principles of XAS as a microspectroscopic technique provides the invaluable opportunity for chemical mapping of metals down to the nanoscale, providing speciation and localisation critical for the mechanistic understanding of the role of metals in specific biological processes.

#### Cryo-soft X-ray tomography (Cryo-SXT)

Cryo-SXT operates on the simple principle that soft X-rays (500 eV) are preferentially absorbed by carbon over water (oxygen) and that in this “water window” region intracellular structures are respectively heterogeneous enough to differentially absorb these soft X-rays to produce distinct contrasts. With its ability to penetrate up to 10 μm, cryo-SXT produces highly resolved (25 nm) 3D reconstructions of the cellular ultrastructure, interactions and complexities of subcellular components such as the nucleus, endoplasmic reticulum, mitochondria, and even parts of the cytoskeletal structure,^56^ within whole cells at near native states, filling a critical resolution gap between electron microscopy and fluorescence imaging.^57^

Although it does not provide metal-specific distribution information as in XRF and XAS, cryo-SXT has been used to visualise metal-based nanoparticle adsorption by cells^58^ and calcium-rich concentrates in algae,^59^ in addition to a whole range of processes including *Plasmodium* heme detoxification,^60^ bacterial activation of T lymphocytes,^61^ degranulation in mast cells,^62^ and chromatin reorganisation.^63^ In a correlative approach with cryo-SXT, immunogold markers, and cryo-fluorescence microscopy, endosomes and autophagosomes, normally negatively affected by sample preparation for electron microscopy, were imaged and the 3D ultrastructure of endocytic and autophagic structures, as well as clusters of omegasomes on the endoplasmic reticulum, which act as a platform for autophagosome biogenesis, were mapped, showing that these powerful approaches in combination can highlight cellular processes inaccessible using other imaging modalities,^64^ providing refined 3D subcellular morphological changes in response to experimental treatments, infection, and even disease in whole unstained cells (Fig. 1C).

#### Coherent diffractive imaging (CDI)

From the elucidation of DNA structure to the structures of proteins, the complex mechanisms of ATP synthesis, and the molecular basis for eukaryotic transcription, X-ray diffraction has played an undeniably pivotal role in deducing 3D structure at the atomic scale and understanding biomolecules. Now new developments in coherent diffractive imaging (CDI) enable 3D structure determination of non-crystalline samples at the nanoscale, opening new areas of imaging capabilities in biology. CDI is referred to as a “lensless” imaging technique, as it avoids the limitations of diffractive X-ray optics by illuminating the sample using a coherent beam and collecting the resulting scattered light, after which the image is reconstructed using an advanced phase retrieval algorithm.^65^ For biological applications, CDI can be viewed as a powerful complement to SRM and electron microscopy (EM), as the use of X-rays allows for large penetration with the ability to image whole biological cells without sectioning, uses the phase shift caused by intrinsic differences in density to obtain quantitative 3D images of intracellular structure, and approaches spatial resolutions obtained by EM by avoiding the use of lenses.^66^ Additionally, CDI can make use of X-ray absorption and provide chemical information, while polarisation can be used to show magnetic contrast and information on molecular orientation, thus allowing for chemical, elemental, and magnetic mapping at the nano-scale.^67^,^68^

One such CDI method that has risen to the forefront of bioimaging is ptychography.^69^,^70^ By using a coherent X-ray probe to scan and collect diffraction patterns from a series of partially overlapping regions in a sample, ptychography can provide detailed image reconstructions at near-wavelength lateral resolution and sub-nm depth resolution (Fig. 1B).^71^ Ptychography, although a relatively new technique, is currently being used in most synchrotron facilities with a wide range of biological applications in the literature. Both cellular structure and distribution of light elements (potassium, sulphur, phosphorus) in the frozen-hydrated green algae *Ostreococcus* sp., were recently imaged with high structural (<30 nm) and elemental composition (90 nm) resolution by combining ptychography and emitted fluorescence signals.^72^ To gain insights into the chemistry and biomineralisation function of magnetosomes (Fe_3_O_4_ nanoparticles) in magnetotactic bacteria (MTB), ptychography was used to image and speciate magnetosomes in *Magnetovibrio blakemorei* MV-1 cells, resulting in a proposed pathway of magnetosome biomineralisation.^73^ Within the context of nanomedicine, two oxidation states of individual iron oxide nanoparticles with a resolution of 16.5 nm internalized within individual HeLa cells were determined,^74^ and for the first time, ptychographic hard X-ray tomography was used to image 100 μm thick frozen biological tissue in 3D, allowing for the visualisation of myelinated axons and age-related pigmented cellular inclusions at 100 nm spatial resolution.^75^ Ptychography is still a relatively new approach but, if current results are any indication, the technique holds great promise for metallomic bioimaging applications going forward.

#### Positron emission tomography (PET)

Positron emission tomography (PET) is known for its clinical use as a functional diagnostic imaging technique to accurately measure the *in vivo* distribution of a range of different radiopharmaceuticals. The most commonly used F-18 fluoro-2-deoxy-d-glucose (FDG) analogue to glucose, whose cellular uptake is directly proportional to glucose metabolism in cells, is significantly increased in malignant tumours, thereby providing the ability to localise malignancy *in vivo*. However, a growing capacity for ^64^Cu, ^63^Zn, ^52^Mn, and ^52^Fe PET is opening the field for *in vivo* metallomics.^76^–^78^ The emergence of a new research front “PET metallomics”, introduced in the literature for the first time several months ago by Bartnicka and Blower, fills a critical gap between our understanding of processes involved in intracellular essential metal homeostasis and how such processes translate to whole body fluxes and transport between different compartments *in vivo* (Fig. 3B).^79^ PET metallomics is certainly in its infancy, but already promising ^64^Cu PET studies are appearing in the literature within the context of Wilson's disease, dementia and cancer, with the potential to improve diagnostics and complement or even replace biopsies.^80^–^84^

## Mass spectrometry imaging

**Fig. 5:**
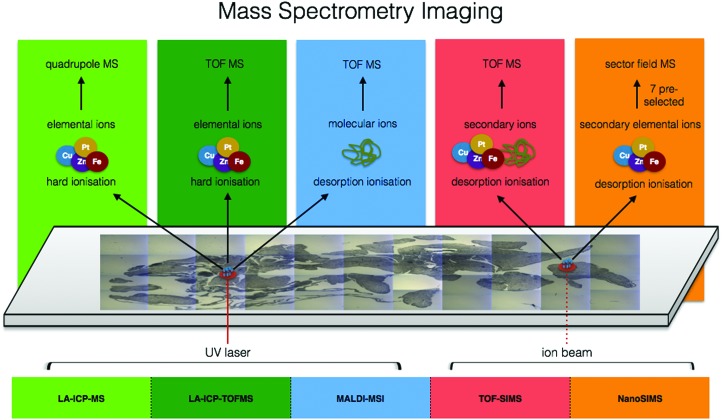
Comparison of ionisation sources, resulting elemental/molecular ions, and mass detectors among mass spectrometry imaging techniques.

Moving from the multitude of analytical possibilities the electromagnetic spectrum offers for *in situ* metallomics, we come to the realm of mass spectrometry. Mass spectrometry imaging (MSI) can be conducted at both molecular and elemental levels and offers several major key capabilities to the metallomic analytical portfolio including spatially resolved molecular identification, isotopic sensitivity, and a wide dynamic range (five orders of magnitude) with the ability to quantify certain elemental isotopes down to parts per quadrillion (ppq) (1 × 10^–15^ g kg^–1^). In the following subsections, several MSI techniques specifically suited for *in situ* analysis of metals in biological samples are discussed. The combination of elemental and molecular mass spectrometry offers unprecedented opportunities for analytical biochemistry (Fig. 5).

### Laser ablation inductively coupled plasma mass spectrometry (LA-ICP-MS)

Of all the metallomic imaging approaches, ICP-MS based techniques have become a quantitative metal “go to” technique for good reason: ICP-MS provides some of the greatest sensitivities with detection limits of 10–100's of ppq for MS/MS systems and even down to single digit ppq for high-resolution magnetic sector field systems, boasts wide dynamic linear range of up to five orders of magnitude, and enables multi-element trace analysis of specific isotopes. These characteristics become critical for metallomic imaging, which requires the analysis of small amounts of biological material containing a wide range of elements in varying concentrations. Within a relatively short period of time, ICP-MS has replaced the previous workhorse of metal analysis, electro thermal atomic absorption spectroscopy (AAS).

With front end coupling of laser ablation systems to ICP-MS, a focused laser beam is used to ablate solid sample material, with the resulting aerosol plume transferred into the ICP *via* a carrier gas. Through the reconstruction of ICP-MS signal counts from time to coordinate space, and with significant developments in the field of laser ablation over the past 10 years, fast generation of isotopically specific elemental distribution maps of biological samples down to spatial resolutions of one micron are opening applications to the cellular level, and sub-micron spatial resolution is now achievable utilising deconvolution methods.^85^ Recently reviewed by Van Malderen *et al.*, new developments in cell ablation technology, faster washout speeds, and advances in post-acquisition image generation software for fast and powerful image rendering and analysis in both 2D and 3D^86^ are supporting an emerging surge of elemental bioimaging utilising LA-ICP-MS as a cornerstone technique.^86^ With additional developments in time-of-flight mass spectrometry (TOFMS), LA-ICP-TOFMS allows simultaneous determination of virtually the entire periodic table. Recent work coupling LA-ICP-TOFMS is incredibly promising for the field of metallomics, as it presents the first opportunity to capture the spatial distribution of the metallome in biological samples. Although ICP-TOFMS is less sensitive than ICP-MS by approximately a factor of 10, very recent advances in ablation cell technology, with introduction of the cobalt cell from Teledyne CETAC Technologies, has brought detection of heavier elements on par with most ICP-MS/MS. The overall result is extremely fast imaging capacity with near simultaneous measurements of the full elemental isotope mass range, supporting an emerging movement towards establishing LA-ICP-MS as a clinical diagnostic tool.

The use of LA-ICP-MS for bioimaging has reached the level of routine analysis in some specialized labs and has been thoroughly reviewed in the literature.^87^ Work is primarily dominated by endogenous mapping of metals in various types of organs (Fig. 2B)^88^–^90^ as well as determining penetration depth of specific metallodrugs,^91^,^92^ and can be done quantitatively using matrix matched standards for calibration. For quantification, a host of solid and liquid standard addition based methods including pressed pellets of reference materials, gelatin standards, metal spiked polymer film and tissue homogenates are thoroughly reviewed by Piekoszewski *et al.*^93^ Decisions on which calibration approach to utilise are taken at laboratory level. Therefore, there is a great need for leading research groups in the field of metallomic imaging to centralise expertise and develop standardised protocols that can be used by the growing metallomics community, key for extending the reach of metallomics and a clear goal for the newly established London Metallomics Facility, discussed at the end of this perspective.

Although tissue-scale analysis is becoming more routine, the increasing need for subcellular resolution, multiplexing capabilities for visualisation of biomarkers and even 3D metal distribution are driving the push past preconceived limitations of LA-ICP-MS with the help of several key researchers and commercial partners in the field. In relatively large fibroblast cells and macrophages, LA-ICP-MS has been used to track gold-labelled human regulatory macrophages in immune deficient mice,^94^ to image iodine as an elemental marker in differentiated fibroblast cells,^95^ the detection of Au NPs within fibroblast cells,^96^ and uptake of cadmium-based quantum dots in HeLa cells.^97^ Yet, many single cell analyses involve significantly smaller cells in the range of 10 μm. Recently, intracellular copper was quantified in the algae *S. trochoidea* by ablating whole individual cells to obtain information on distribution of copper uptake within the population as well as subcellular imaging using a spot size of 2 μm. Most importantly, this study cross-evaluated results with those obtained by SXRF, showing similar accumulation patterns and being the first to demonstrate the potential of LA-ICP-MS, not only for bioimaging at the subcellular level, but also its potential for high throughput single cell analysis.^98^ The increasing demand for establishing and imaging biomarkers within the context of human disease has led to multiplexing approaches in which metal lanthanide tagged antibodies are used to image specific proteins in tissue sections. Pioneering work illustrated this capability by imaging HER2 and 32 other proteins in human breast cancer tissue down to 1 μm spatial resolution,^99^,^100^ and this approach is now being explored in single cell imaging applications.^101^ Very recent software advances in post acquisition data processing and multi-modal data fusion have opened new possibilities for 3D LA-ICP-MS,^102^ demonstrated in a handful of publications looking at metals in murine brain with a spatial resolution of 80 μm,^103^ in mature wheat and rye grains with a spatial resolution of 20 μm,^104^ and most recently in *Ceriodaphnia dubia* with a spatial resolution of 5 μm using LA-ICP-TOFMS, correlated to microcomputed tomography (μ-CT).^102^ 3D LA-ICP-MS has been time consuming, not only in data acquisition but also in data processing and image generation. However, as a result of recent combined hardware and software advances (HDIP software), 3D LA-ICP-MS is likely to play a greater role in the context of correlative 3D imaging.

### Secondary ion mass spectrometry (SIMS)

Secondary ion mass spectrometry (SIMS) is by no means a new technique, being used for over half a century for surface analysis of inorganic materials. However, SIMS has undergone a revival with newly developed bioimaging applications, as it offers the highest lateral resolution of any MSI technique and is capable of imaging chemical species in tissues and single cells, due to its ability to achieve sub-micron spatial resolutions for both element and molecular imaging in the mass range from 1–2000 Da.^105^ In SIMS, a focused ion beam bombards a sample under vacuum, resulting in the ejection and ionisation of molecules from the sample surface. These ejected secondary ions are then analysed in a mass spectrometer. It is important to note that the majority of ejected molecules are in fact neutral and ionisation is not uniformly efficient for all species, illustrating one important caveat in SIMS with respect to metallomic applications involving transition metals that tend to ionise less easily relative to lighter elements. Despite these ionisation challenges, SIMS is still used to visualise metals in biological samples and adds the additional benefit of imaging lighter elements and molecular fragments to provide an informative molecular context for metals. SIMS can be run in both dynamic and static modes, where dynamic mode allows for depth profiling and excessive fragmentation leads to excellent elemental analysis, and where static mode minimizes surface erosion providing surface-sensitive molecular information.^106^ Although SIMS is not normally operated quantitatively with biological samples due to large variations in both sputtering efficiency (from heterogeneity of sample) and the ion yield of an element (from differences in inherent ionisation efficiencies), methods have been established to do so.^93^ Lighter elements are more readily ionised and thus SIMS is typically used to look at local intracellular concentrations of elements such as calcium, potassium, magnesium, and sodium.^107^

Within the context of bioimaging, three main types of SIMS instruments have risen to the forefront: Fourier Transform ion cyclotron resonance (FTICR) SIMS, with its highly accurate mass-resolving power, time-of-flight SIMS (TOF-SIMS), chemically mapping samples at an order of magnitude better spatial resolution that FTICR-SIMS and with the additional benefit of depth profiling, and NanoSIMS, which offers sub-100 nm spatial resolution (Fig. 1D).^108^ A few examples include the use of NanoSIMS to visualize association of copper with cell components rich in proteins and phosphorus, highlighting the use of SIMS for intracellular tracking of essential trace elements in single cells.^109^ NanoSIMS showed the increase of ferritin in the coronal region of human AD plaques,^110^ whereas TOF-SIMS quantified cholesterol overload in the cerebral cortex of AD patients.^111^

### Matrix assisted laser desorption/ionisation mass spectrometry imaging (MALDI-MSI)

Whereas SIMS is typically limited to a narrow mass range window of a few hundred Daltons, MALDI-MSI can expand this range and image molecules as large as 70 kDa in some instances,^112^ providing quantitative^113^ information on a heterogeneous molecular landscape for a wide range of analytes spanning proteins, peptides, and lipids to drugs and their metabolites.^114^ Despite MALDI-MSI being a label free approach, samples must be embedded in a chromophore-containing matrix to assist in the photo-volatilisation and ionisation process, which occurs as a result of a UV laser beam rastered across the sample surface under atmospheric pressure. The resulting ionised material is typically transferred to a TOF mass analyser, but higher mass resolving power can be obtained when coupled to an Orbitrap^115^ or FTICR mass analyser, allowing for protein identity assignment of similar average mass and with interspersed isotopomers revealing the presence of many protein adducts.^116^ With recent advances, spatial resolutions down to 1 μm are now possible, however 10–20 μm are more commonly used making this technique most appropriate for tissue level analyses.^114^

Direct imaging of metal-containing compounds using MALDI-MSI is challenging due to poor ionisation efficiency and signal suppression from biological matrix effects.^117^ Therefore, MALDI-MSI is typically used in conjunction with LA-ICP-MS on sequential tissue sections and images are correlated in a “guilt by association” strategy (Fig. 2B). However, despite these analytical challenges, using an optimized sample preparation protocol, oxaliplatin and its metabolites were mapped in multicellular tumour spheroids for the first time, showing that most of the drug was detected in the outer area, and oxaliplatin complexed to methionine was at the core.^118^ When MALDI-MSI utilises high resolution mass spectrometry, the high mass resolving power of these techniques should allow for the identification of the molecular formulae including predictions of associated metals.^1^

## Particle beam microscopy

**Fig. 6:**
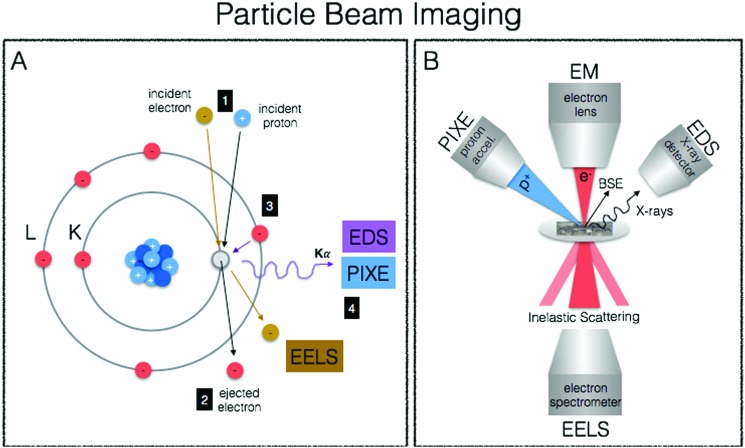
Principles of particle beam imaging techniques. (A) In both EM-EDS/EELS and PIXE a particle (electron or commonly proton, respectively) with sufficient energy is used to eject a core electron (1). In EM-EELS, the inelastically scattered incident electron is measured and the corresponding loss of energy provides element specific information such as coordination geometry and oxidation state (2). When an outer shell electron fills the inner core hole (3), element specific X-ray emission occurs and is measured in both EM-EDS and PIXE (4) to reveal elemental composition of a sample. (B) A schematic representation illustrating the principles of particle beam imaging techniques (BSE = backscattered electron).

We end our journey into the physical interactions of energy and matter with particle beam microscopy, where resulting physical phenomena from particle–matter interactions give rise to high-resolution images well beyond the diffraction limit of visible light, provide elemental information and chemical mapping, and fully quantitative elemental localisation (Fig. 6A, B). Taking advantage of the particle/wave dualism of the electron, its short wavelength (0.004 nm) allows for sub-nm spatial resolution of electron microscopy, providing unprecedented capabilities for obtaining subcellular structural context. When coupled to energy-dispersive (EDS/EDX) X-ray detectors and/or detectors for electron-energy loss spectroscopy (EELS), elemental identification and speciation can be incorporated. Particle induced X-ray emission (PIXE) provides increased sensitivity and fully quantitative elemental mapping, albeit at less refined spatial scales. Together these techniques fill important gaps in spatial resolution required for biological structural contexts as well as absolute quantitative mapping of metals down to the cellular level.

### Electron microscopy (EM)

The best known particle-based imaging approach is no doubt electron microscopy (EM), operating on the same basic principles as light microscopy, but instead taking advantage of the short wavelength of electrons to achieve resolution many orders of magnitude better to reveal the finest details of internal structure (Fig. 1D), down to individual atoms in some cases. It is of no surprise then that EM has developed into an important cornerstone of biological imaging to address questions at the most refined spatial scales. Transmission EM (TEM), scanning transmission EM (STEM) and scanning EM (SEM) are used for analysis of biological material, major differences difference being that TEM/STEM sections are typically less than 100 nm, measure transmitted electrons, and offer the highest spatial resolution (down to 0.5 Å). In SEM, relatively thicker sections are used making sample preparation easier, and backscattered electrons are measured resulting in a loss of spatial resolution (0.4 nm) but increased depth of field. With advancements in cryo EM and developments in milling for serial block face imaging using focused ion beam (FIB), it is now possible to use cryo FIB-SEM for the direct and fast 3D imaging of large native frozen samples, including tissues.^119^

TEM and SEM are not only used for generation of highly resolved ultrastructure, but when coupled EDS X-ray detectors and/or detectors for EELS, surface elemental composition can be obtained. When this analytical information is presented as a false colour overlay on traditional EM images, ions and biomolecules are directly visualised and elemental fingerprints of subcellular components are analysed in an approach coined as ColorEM.^120^ EDS and EELS are complementary in that EDS is particularly sensitive to heavier elements up to molybdenum, whereas EELS is best used for lighter elements ranging from carbon through the 3d transition metals. An additional benefit of EELS is the ability to image structure through carbon, nitrogen, and oxygen without counterstaining and speciation information of the coordination environment is possible. Although (S)TEM/SEM-EDS/EELS can provide the highest spatial resolutions, element detection is orders of magnitude less sensitive than compared to X-ray spectroscopic and mass spectrometric techniques due to intense Bremsstrahlung background, particularly for EDS detection, and applications in metallomic bioimaging have been recently reviewed.^121^

STEM-EELS is the preferred EM method for trace metal mapping in biological samples due to its increased sensitivity relative to EDS.^122^ Using STEM-EELS calcium concentrations in the endoplasmic reticulum and mitochondria were mapped in 100 nm thick sections of mouse cerebellar cortex, indicating the role of the endoplasmic reticulum in calcium regulation,^123^ and quantitative manganese mapping of rat brain cells showed that manganese particles primarily localised in mitochondria.^124^ This approach has also been extended to 3D with post acquisition computational methods, as illustrated in the generation of 3D maps of iron bound to ferritin in degenerating neurons of mice^125^ and new applications for the identification of markers and nanomaterials within the context of diagnostics and therapeutics have recently emerged.^121^

### Particle-induced X-ray emission (PIXE)

Although a far cry from the spatial resolution capabilities of TEM/SEM-EDS/EELS, μ-PIXE is more sensitive and concentrations are fully quantitative without the need for calibration as particle beam interactions with electronic shells of the atoms have been extensively described.^126^ μ-PIXE is based on the same physical excitation phenomenon previously described for X-ray spectroscopy, with the difference being the source of excitation (protons in many cases). When carried out in combination with RBS (Rutherford Backscattering Spectrometry) to measure mass of sample, metal content can be spatially expressed in terms of weight of element per g of dry mass,^127^ with the powerful effect of being able to correct for changes in material density within a given sample. With spatial resolutions down to 1 μm and good sensitivity for essential trace elements (*i.e.* manganese, iron, copper, zinc, selenium), toxic heavy metals (mercury, lead) and pharmacological compounds (platinum), μ-PIXE provides a useful tool for metal distribution studies at the subcellular level.^126^

For example, μ-PIXE showed that cobalt accumulated in the nucleus and peri-nuclear regions of skin human cells, causing a decrease in both magnesium and zinc and suggesting that cobalt may interfere in magnesium and zinc cellular homeostasis.^128^ Gadolinium-based nanoparticles along with essential elements (phosphorus, sulphur, chlorine, potassium, calcium, iron, copper, zinc) were quantitatively mapped in U87 human glioblastoma tumours with high spatial resolution using a combination of μ-PIXE and RBS, showing heterogeneous gadolinium NP distribution and effects to endogenous elemental distribution and concentrations (Fig. 2A).^129^

## Multidimensional metallomics

At the heart of our metallomic investigations we ideally seek to quantify metal species in a particular location within a meaningful biological/functional context at a certain point in time. In reality, the ability to obtain this information in a single measurement is virtually impossible. We frequently face challenges of low concentrations in complex biological matrices often with the requirement for high spatial resolution and perhaps even measurement of temporal dynamics. Because of these circumstances, there is a clear need to use and continually develop new correlative workflows between analytical techniques that can provide complementary information to address questions of metal quantification, localisation, chemical and structural biological contexts, and temporal dynamics in metal- related biological processes. This statement is not new. Certainly we have read the growing number of calls in the literature for more correlative based approaches in science. However, even in reading state of the art reviews, one can get easily lost in a sea of acronyms, and despite reading countless articles, it feels quite a daunting task to have a comprehensive understanding of how such approaches can be combined and what each has specifically to offer.

I have always envisioned a “go to” metallomic analytical table that finally presented the best selection of analytical tools for *in situ* metallomic studies. In this light, Table 1 presents a synthesis of techniques described in this perspective, meant to be an overview reference guide, which can be utilised in a multifaceted way. Rows display information on what each technique provides, grouped according to spectroscopy, spectrometry and particle beam approaches, each with corresponding literature citations of comprehensive reviews addressing specifics such as sample preparation and calibration. Columns allow for quick identification of techniques within the context of a specific requirement (*i.e.* quantification *vs.* speciation *vs.* localisation of metals). Additional colour coding allows for easy identification of techniques best suited for the level of study (*i.e.* subcellular, whole cell, tissue, organ). It should also be noted that often sophisticated and/or expensive instrumentation is required to conduct the analyses described, thus requiring use of core facilities and/or collaboration with specialised research groups as well as the pursuit of access calls at synchrotron facilities worldwide. Despite such approaches being far from fast bench-top solutions, the benefits of the required cross-disciplinary interactions speak for themselves in the results produced.

**Table 1:** Matrix of *in situ* metallomic analytics. A summary of described techniques are presented and organised according to spectroscopy, mass spectrometry and particle beam based techniques for comparison of limits of detection, metal speciation capabilities, best possible spatial resolutions, structural contexts, and dynamic capabilities. Colour coding corresponds to the level of appropriate biological application categorised at whole body (red), tissue (green) and (sub)cellular (blue). Numbers correspond to citations and letters denote specific application notes

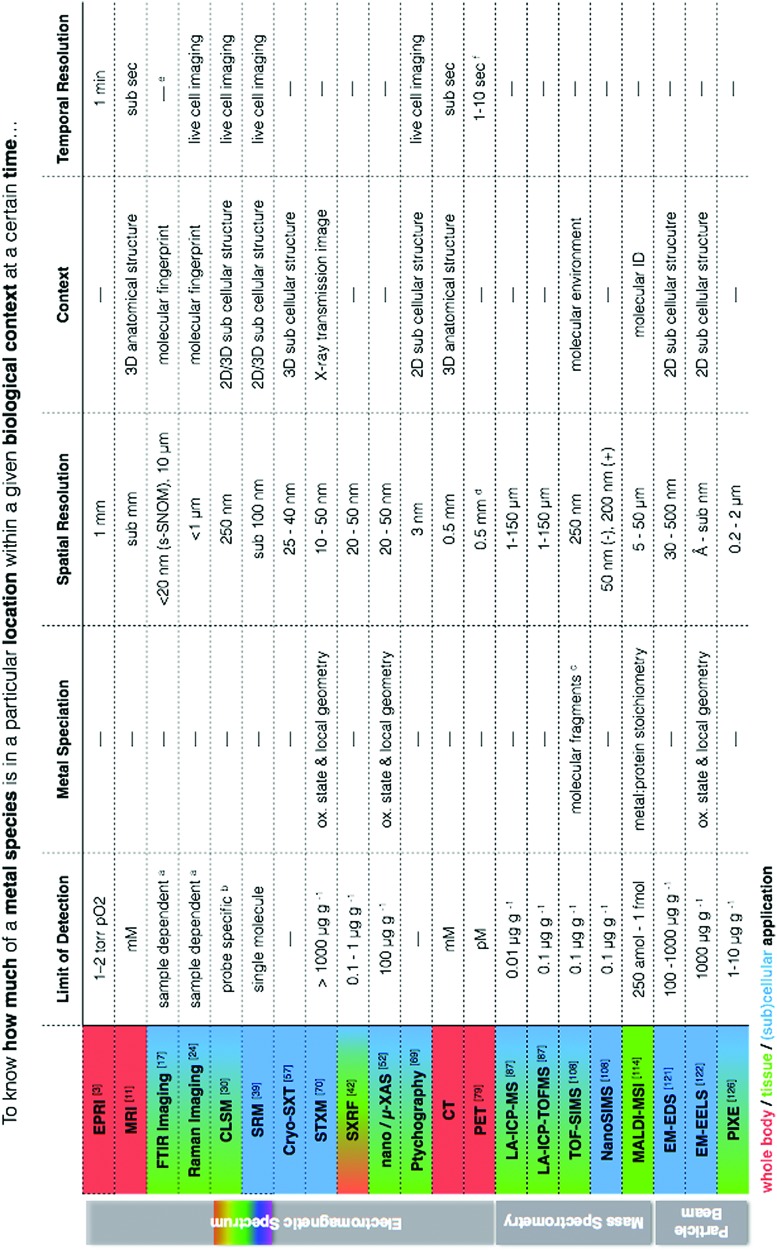

^*a*^LOD for FTIR and Raman spectroscopy depends on concentration, sample thickness, and background interfering spectral features (*i.e.* S/N).

^*b*^LOD dependent on the specificity and selectivity of the probe or sensor.^30^ For zinc, pM concentrations are detectable intracellularly.^138^

^*c*^A low primary ion beam current is used liberate ions, molecules and molecular clusters for analysis so that characterisation of organic macromolecules is possible, in contrast to NanoSIMS, which is better for quantitative analysis of elements due to a higher primary ion beam current and higher secondary ion yield but with a loss of organic compounds.

^*d*^Resolution is 0.5 mm in animals and 0.5 cm in humans.

^*e*^Typically performed on fixed or dehydrated samples due to H_2_O interferences, but live cell imaging at synchrotron facilities using microfluidic devices with D_2_O have been performed.^139^

^*f*^Scanning speeds can be decreased to less than 1 s if activity is high enough.

Correlative workflows should provide complementary information, resulting in a greater understanding of a question at hand, which could not have been otherwise answered. This goal is different than using multiple techniques to confirm a specific observation (*i.e.* using μ-XRF and LA-ICP-MS to localise a metal at similar spatial resolutions). This statement is not meant to discredit the immense value and power of confirming an observation using multiple approaches, but rather to highlight the important distinction between corroboration and correlation of different information (*i.e.* speciation and localisation) to gain a richer understanding.

Recently, the linkage between bone crystallinity and ratios of Mg/Ca and Sr/Ca was explored in two rat models with chronic kidney disease (CKD) and diabetes mellitus (DM) by combining LA-ICP-MS, Raman spectroscopy and SEM. Higher Mg/Ca ratios, determined using LA-ICP-MS, correlated to a decrease in bone crystallinity, measured using Raman, and indicated calcium replacement by magnesium in deterioration of the hydroxyapatite (HAp) structure. To further investigate the change in crystallinity, SEM was used to measure HAp crystals, which decreased with increasing Mg/Ca and Sr/Ca. Interestingly, both crystallinity and Mg/Ca ratios for CKD rats were significantly different from controls and DM rats, highlighting that possible disease related alterations in elemental metabolism may influence ionic substitutions of bone calcium.^130^ Within the context of anti-cancer therapies, correlations between changes in protein secondary structure and platinum distribution were discovered, as well as between cell nuclear morphology and decrease of phosphorus, zinc, and magnesium through combining LA-ICP-MS and FTIR spectroscopy. Multivariate analyses of the results indicated different degrees of tumour viability and successfully discriminated dead tumour regions.^131^ Within the context of exploring the use of magnetic nanoparticles for diagnostic imaging, therapeutics and biotechnology, the uptake and interaction of Ag–magnetite and Au–magnetite composite nanoparticles in 3T3 fibroblast cells was studied. Cryo-SXT confirmed endocytotic uptake and stability of particles in endosomes and lysosomes, indicating their potential use as both optical and magnetic probes in living cells. SERS provided information on the local chemical environmental of the particles, indicating a change in surface composition and interaction of intracellular biomolecules specifically with the magnetite. Comparison of nanoparticle uptake and localisation with and without magnetite using LA-ICP-MS indicated that the magnetite is an important component for uptake.^58^ In another study, NanoSIMS allowed chemical imaging of macro and trace elements with subcellular resolution (element mapping). Calcium, magnesium, and phosphorus as well as the trace elements iron, copper, and zinc present at basal levels were detected in pyrenoids, contractile vacuoles, and granules. Some metals were even localized in small vesicles of about 200 nm size.^132^ In remarkable work, a conceptual model of the coccolith calcium pathway, essential for understanding coccolithophore calcification process within the global carbon cycle, was proposed based on results from a combination of techniques spanning light microscopy, X-ray spectroscopy and electron microscopy. Combining cryo-SXT, spatially and temporally resolved XANES, cryo-focused ion beam scanning electron microscopy (FIB-SEM), EDX and CLSM, a highly concentrated and previously unidentified pool of intracellular calcium was both imaged and speciated within the ultrastructural environment and showed that polyphosphates and other elements including magnesium, are co-localized with calcium.^59^ This work in particular highlights how a comprehensive picture of an important biological process can be ascertained when a wide range of analytical techniques are combined.

## Conclusion & future directions

The field of metallomics takes a holistic approach to understand the metallobiochemistry of cells and organisms by utilising analytical tools to decipher the biological roles of metals in both space and time.^133^ Every cell utilizes a remarkably diverse range of metal ions (sodium, potassium, magnesium, calcium, iron, manganese, copper, zinc, and molybdenum) and non-metals (selenium, chloride, bromide and iodide) in addition to the standard elemental building blocks of biological molecules (carbon, oxygen, nitrogen, hydrogen, sulphur and phosphorus). The *in situ* metallomic imaging tools described in this perspective have the power to characterise these metallomes within the life span of individual cells and tissues with significant value for fundamental scientific questions relating to determination of cell fate and health, trace element metabolism, and for translational science in diagnosis, prognosis, and therapy. In fact, metallomes are as dynamic as the biological systems they exist within, opening another dimension of metallomics called meta-metallomics that addresses individual variation in the life trajectory as well as the influence of environmental factors such as nutrition, exposure and disease.^134^ There is currently an unmet recognized scientific need for establishing such profiles, and through the integration of state of the art analytical techniques described in this perspective, we can now generate elemental profiles with both spatial and temporal resolution within functional biological contexts at scales ranging from atomic to whole body.

Not only is a more comprehensive utilisation of correlative techniques required to advance the field of metallomics, but, more significantly, a shift in our thinking and experimental approach in employing and applying these techniques. When I step back in life and summit that peak to get a full 360 degree view, magically everything's proper order and function becomes clear. Science is not isolated from life (quite the contrary), and this simplistic yet powerful clarity is blurred when our natural scientific tendency towards exclusivity dominates our thinking. From my many conversations with chemists, biologist, physicists, bioinformatisticians, engineers, and even clinicians from fields ranging from stem cell biology to global nutritional health to atmospheric pollution and environmental toxicology, one point is absolutely clear: there is a growing recognition that we cannot expect to solve our pressing scientific problems by summing up fragmented pieces of information obtained from the old “silo” approach. Across all disciplines, there is an overwhelming call in the literature for more integrative experimental approaches and analytical techniques to foster comprehensive cross-disciplinary research. To be able to put each analytical tool in its proper perspective and, rather than seeing individual disconnected methods, see a powerful and integrated network of connected approaches is the future trend, not only for the field of metallomics but science as a whole. What if we were to take a cue from Nature, where nothing operates under exclusivity and perfectly functions in an all-encompassing dynamic inclusivity. To fully understand the multitude of questions and be able to present real solutions for human health and disease along with our environmental impact, like Nature, we must also operate in a multiple and all-inclusive fashion in our experimental and analytical approach. The analytics must be placed into a larger context, and this perspective piece illustrates the multitude of synergistic capacities within a metallomics methodological approach, where with imagination, intuition and focused interaction between researchers from different fields, metallomics can grow to stand with the likes of genomics, proteomics and metabolomics.

To accomplish this goal, a scientific platform is required on which direct and productive interactions between researchers in all scientific fields, clinicians, and commercial partners are established. It requires us as scientists to transcend the present structure of education and research disciplines. With the recent creation of the London Metallomics Facility (LMF), finally comes the incredible opportunity to establish such a platform, which I am honoured to play a role in shaping. The vision of the LMF is not only to expand frontiers in correlative metal bioimaging through offering the most advanced-to-date portfolio of metallomics approaches to address the structural and functional roles of chemical elements in biology and medicine, but to also serve as a centralising hub to comprehensively integrate current analytical expertise in metallomics, foster direct interaction between leading research groups, and lead educational outreach and public engagement strategies. In this sense, metallomic analytics is more than about understanding the role of metals in biology. It presents a powerful opportunity to show in action how we can do science more effectively in a highly collaborative way.

## Abbreviations

*p*O_2_: Oxygen partial pressure
μ-CT: Microcomputed tomography
AAS: Atomic absorption spectroscopy
AFM: Atomic force microscope
AS: Alzheimer's disease
CDI: Coherent diffractive imaging
CKD: Chronic kidney disease
CLSM: Confocal laser scanning microscopy
Cryo-SXT: Soft X-ray tomography
DM: Diabetes mellitus
EDS/EDX: Energy-dispersive X-ray spectroscopy
EELS: Electron-energy loss spectroscopy
EM: Electron microscopy
EPRI: Electron paramagnetic resonance imaging
ESR: Electron spin resonance
EXAFS: Extended X-ray absorption fine structure
FIB: Focused ion beam
FRET: Förster resonance energy transfer
FTICR: Fourier transform ion cyclotron resonance
FTIR: Fourier Transform infrared
HAp: Hydroxyapatite
LA-ICP-MS: Laser ablation inductively coupled plasma mass spectrometry
MALDI-MSI: Matrix assisted laser desorption/ionisation mass spectrometry imaging
MRI: Magnetic resonance imaging
MRM: Magnetic resonance microscopy
MSI: Mass spectrometry imaging
MS: Multiple sclerosis
PALM: Photo activated localisation microscopy
PET: Positron emission tomography
PIXE: Particle induced X-ray emission
RBS: Rutherford backscattering spectrometry
ROS: Reactive oxygen species
RRS: Resonance Raman spectroscopy
TEM: Transmission electron microscopy
TOFMS: Time-of-flight mass spectrometry
SEM: Scanning electron microscopy
SERS: Surface enhanced Raman spectroscopy
SIM: Structured illumination microscopy
SIMS: Secondary ion mass spectrometry
SMLM: Single molecule localisation microscopy
SPIM: Selective plane illumination microscopy
SR: Synchrotron radiation
SRM: Super resolution microscopy
SRS: Spectroscopic stimulated Raman scattering
s-SNOM: Scanning near-field optical microscopy (s-SNOM)
STED: STimulated emission depletion microscopy
STEM: Scanning transmission electron microscopy
STORM: Stochastic optical reconstruction microscopy
STXM: Scanning transmission X-ray microscopy
SXRF: Synchrotron X-ray fluorescence
XANES: X-ray absorption near edge structure
XAS: X-ray absorption spectroscopy

## Conflicts of interest

There are no conflicts to declare.
